# Strengthening and monitoring health system's capacity to improve availability, utilization and quality of emergency obstetric care in northern Nigeria

**DOI:** 10.1371/journal.pone.0211858

**Published:** 2019-02-06

**Authors:** Ibrahim Kabo, Nosa Orobaton, Masduk Abdulkarim, Emmanuel Otolorin, Toyin Akomolafe, Dele Abegunde, Emma Williams, Habib Sadauki

**Affiliations:** 1 Targeted States High Impact Project (TSHIP), Jhpiego, an affiliate of Johns Hopkins University, Bauchi, Nigeria; 2 Targeted States High Impact Project (TSHIP), JSI Research and Training Institute, Bauchi, Nigeria; 3 Jhpiego, Baltimore, MD, United States of America; University of Heidelberg, GERMANY

## Abstract

**Background:**

Quality improvement in emergency obstetric care (EmOC) is a critical and cost-effective suite of interventions for the reduction of maternal and newborn mortality and morbidity. This study was undertaken to evaluate the impact of quality improvement interventions following a baseline assessment in Bauchi state, Nigeria.

**Methods:**

This was a prospective before and after study between June 2012, and April 2015 in Bauchi State, Nigeria. The surveys included 21 hospitals designated by Ministry of Health (MoH) as comprehensive EmOC centers and 38 primary healthcare centers (PHCs) designated as basic EmOC centers. Data on EmOC services was collected using structured established EmOC tools developed by the Averting Maternal Death and Disability (AMDD), and analyzed using univariate and bivariate statistical analyses.

**Results:**

Facilities providing seven or nine signal EmOC functions increased from 6 (10.2%) in 2012 to 21 (35.6%) in 2015. Basic EmOC facilities increased from 1 (2.6%) to 7 (18.4%) and comprehensive EmOC facilities rose from 3 (14.3%) to 13 (61.9%). Facility birth increased from 3.6% to 8.0%. Cesarean birth rates increased from 3.8% in 2012 to 5.6% in 2015. Met need for EmOC more than doubled from 3.3% in 2012 to 9.9% in 2015. Direct obstetric case fatality rates increased from 3.1% in 2012 to 4.0% in 2015. Major direct obstetric complications as a percent of total maternal deaths was 70.9%, down from 80.1% in 2012.

**Conclusion:**

The rise in the percent of facility-based births and in met need for EmOC suggest that interventions recommended and implemented after the baseline study resulted in increased availability, access and utilization of EmOC. Higher patient load, late arrival and better record keeping may explain the associated increase in case fatality rates.

## Introduction

The burden of maternal deaths remains substantial in many developing countries [1]. In 2013, there were an estimated 289,000 maternal deaths globally, equivalent to a decline of 45% from 1990 [2]. Developing countries accounted for 99% (286,000) of the global maternal deaths with the Sub-Saharan African region accounting for 62% (179, 000), followed by Southern Asia with 24% (69 000) [2]. Nigeria accounted for 14% (approximately 40, 000) of all global maternal deaths. While there has been a reduction in the global maternal mortality ratio (MMR) between 1990 and 2013, Nigeria’s MMR of 576/100,000 live births in 2013 [3] remains more than 2 times higher than the MMR of 230/100,000 live births in developing regions and 36 times higher than the MMR of 16/100,000 live births in developed regions [2]. Not only is the MMR in Nigeria higher than the 510/100,000 live births recorded in the Sub-Saharan African region, the adult lifetime risk of maternal mortality in women was 1 in 38 in 2013 compared to only 1 in 3700 among women in developed countries [2].

Despite the steep decline in the MMR in Nigeria from 800 deaths per 100,000 live births in 2003 to 576 in 2013, the rates themselves remain very high compared to the rest of the world [3–5]. Therefore, Nigeria was not able to achieve the target set by the Millennium Development Goals (MDG) to reduce MMR to 250 deaths per 100,000 live births by the year 2015 [6].

Results from the 2013 Nigeria Demographic and Health Survey (NDHS) showed a decline in infant mortality from 100 deaths per 1,000 live births in 2003 to 69 in 2013, while neonatal mortality declined from 48 in 2003 to 37 deaths per 1,000 live births in 2013. The results thus far attained are far behind the targets of the MDG set to reduce infant mortality to 30 deaths per 1,000 live births by 2015 [6,7].

Utilization of Maternal and Newborn Health (MNH) services in Nigeria has remained relatively stagnant over the last 10 years. Childbirth attended by a skilled birth attendant and in a health care facility each increased by only three percentage points from 35% in 2003 to 38% in 2013 and from 33% in 2003 to 36% in 2013, respectively. Births occurring at home decreased by only three percentage points during the 10 year period from 66% in 2003 to 63% in 2013 [3].

The care a woman receives during pregnancy, childbirth, and the immediate postnatal period is essential not only to ensure the normal, healthy evolution of the pregnancy but also to prevent, detect or predict potential complications during pregnancy, childbirth, and the postpartum period. Thus quality of care is essential for the survival and well-being of the woman and her newborn [1,3]. Good quality care must be provided by skilled health professionals adequately equipped to prevent or detect potential complications, and provide the necessary lifesaving intervention or referral [1].

Availability, utilization and quality of emergency obstetric care (EmOC) services in Bauchi state, Northeast Nigeria is below the United Nation’s (UN) minimum standard while the recorded MMR and neonatal mortality rate are among the highest in the country [8]. An estimated 2,000 maternal and 12,000 neonatal deaths take place in Bauchi state annually [3]. The utilization rate of MNH services in Bauchi is one of the lowest in Nigeria. Although 55.8% of women in the state received antenatal care from a skilled provider, only 16.9% gave birth in a health care facility. Eight out of every ten women in the state give birth at home and 21.2% of them give birth alone with no one present to offer assistance during childbirth [3].

Improving the availability, accessibility, utilization and quality of essential evidence-based maternal health interventions, including EmOC, with particular consideration to both comprehensive and basic EmOC, are critical to the reduction of maternal mortality and morbidity, particularly in low-income settings [9–14].

Between June and July 2012, the Bauchi State Ministry of Health (MOH), with the support of the Targeted States High Impact Project (TSHIP), conducted a baseline, cross-sectional survey of 21 general hospitals designated as comprehensive EmOC (CEmOC) and 38 primary health care centers designated as basic EmOC (BEmOC) across the 3 senatorial zones in the state. Each senatorial zone consists of six to seven local government authorities (LGAs). The goal of the survey was to assess the availability, utilization, and quality of EmOC services in Bauchi State [8]. The baseline survey found a number of indicators of poor quality services and poor patient outcomes.

The survey showed that only 6 (10.2%) of the 59 facilities met the UN requirements for EmOC centers and only 3 (15.0%) of the hospitals were fully functioning CEmOC facilities. None of the three senatorial zones in the state met the UN’s recommendation of five EmOC facilities per 500,000 people. The findings also indicated that overall, only 4% of the statewide estimated annual births took place in EmOC facilities. Cesarean births accounted for 3.6% of births occurring in EmOC facilities and only 0.2% of expected live births. The direct obstetric case fatality rate in EmOC facilities was 3.2%, with only 3.9% of the expected obstetric complications being managed in EmOC facilities.

Bauchi State MOH with support from TSHIP and other stakeholders used these findings to develop and implement seven specific categories of intervention from 2012 to 2015.

This paper describes the evaluation of these interventions, demonstrating progress made since the 2012 baseline survey. The assessment examined the recommended intervention areas that were implemented, namely health facility improvement, competency-based training, provision of equipment, supportive supervision, demand generation, and quality improvement. The paper is also an account of lessons learned, issues and challenges that remain and suggestions for refinements to current efforts, to improve the quality of EmOC services in Bauchi State.

## Methods

### Design

This was a prospective intervention study of EmOC services using a pre and post intervention design between June 2012, and April 2015 in Bauchi State, Nigeria. The study was conducted to evaluate interventions to improve the access and quality of EmOC services and reduce maternal mortality and morbidity. Baseline data were collected between June and July 2012. A follow-up assessment was conducted between March and April 2015. The study used methodologies and data collection instruments identical to those employed in 2012.

The following interventions were implemented by Bauchi State MOH with support from TSHIP and other stakeholders between 2012 between 2015 based on the recommendations from the baseline survey.

### Health facility improvements

TSHIP supported renovations and minor repairs at hospitals and PHCs to increase and improve their readiness to respond to obstetric emergencies. A total of 10 hospitals and 14 PHCs among the surveyed health facilities were renovated. The government assessed and commenced renovation of additional 7 hospitals and 23 PHCs and health centers to increase access and utilization.

### Provision of essential equipment and supplies

TSHIP worked with Bauchi state to supply missing essential equipment for the labor and delivery room, antenatal and postnatal wards, and operation rooms for all the surveyed health facilities. This provided opportunity for the trained healthcare workers to practice competencies learnt during trainings for provision of quality EmOC services.

### Capacity building

TSHIP worked with the MOH to conduct training-of-trainers for 20 health professionals and these conducted several stepdown competency-based training activities (CBTs) to equip doctors, midwives and community health extension workers (CHEWs) with the knowledge and skills to provide safe care during labor and childbirth, prevent, detect and manage/refer obstetric complications. The trainers continued to serve as state trainers for subsequent state organized trainings to reach more health facilities.

### Supportive supervision and quality improvement

TSHIP worked with the MOH to strengthen the integrated supportive supervision (ISS) system at state and local government levels. It also facilitated arrangements that would enable various components within the health sector to work together to make supervision more integrated and supportive for better performance and improved quality of health care services. State and local government authority (LGA) supervision teams were formed, trained and equipped to conduct ISS at hospitals and PHCs on a quarterly basis. Zonal meetings of ISS teams were introduced and were held bi-annually to share experiences, lessons learned and to develop strategies that addressed challenges. This strengthened the state and LGA teams to plan, implement ISS and monitor progress. The state continued to conduct ISS services after the TSHIP project ended.

### Provision of job aids and protocols

TSHIP worked with MOH and other stakeholders to develop learning resource materials for MNH services. These included job aids/protocols on prevention and management of postpartum hemorrhage (PPH), Essential Newborn Care (ENC), care of the preterm and low birth weight babies, newborn resuscitation, management of severe pre-eclampsia and eclampsia, focused antenatal care (FANC), and care of the umbilical cord, among others. Bauchi state re-printed these job aids and distributed to other health facilities.

### Improving record keeping and documentation

Prior to TSHIP, Bauchi State’s health management information system used inadequate and inconsistent data reporting registers with poor availability of data at the Local Government Council and State levels. The Bauchi State Ministry of Health was supported to establish a multi-sectorial health data consultative committee (HDCC), responsible for cooperation, collaboration and coordination of health information system especially in the area of health data collection, flow, custody and release/disseminations. This led to improved data collection, reporting and quality.

### Demand generation and community engagement

TSHIP worked with the MOH to re-activate ward development committees (WDCs) and trained community-based health volunteers (CBHVs), most of whom are traditional birth attendants (TBAs) to counsel households on the importance of ANC, childbirth in a health care facility, personal hygiene, breastfeeding, maternal and newborn danger signs, child spacing, use of misoprostol for prevention of bleeding after birth and chlorhexidine for prevention of umbilical cord infection. This was critical for improving the health seeking behavior of pregnant women and their families. Bauchi state has continued to procure misoprostol and chlorhexidine for community distribution and is supporting the CBHVs program across the state.

### Study sites

Data for the baseline and endline surveys were obtained from 21 hospitals and 38 primary healthcare centers in Bauchi State. All the facilities studied were public facilities located in the state’s three senatorial zones that provide labor and childbirth services. Included in this survey were the clinical departments within each given facility that provided maternal and newborn care services. Of the 23 general hospitals in the state, two were excluded because they were inaccessible owing to insecurity. The primary healthcare centers administratively clustered around the included hospitals were sampled from the 318 primary healthcare centers distributed across the state.

### Data collection

Data were collected in the baseline and follow-up surveys using EmOC evaluation tools developed by Averting Maternal Death and Disability [8]. The tools were based on the EmOC indicators specified in the international guidelines for monitoring use of maternal and neonatal services [7]. An identical modular questionnaire was adapted in 2012 and covered provider knowledge and competency for maternal and newborn care, EmOC signal functions, cesarean births, and maternal deaths, was used as well.

Twenty-seven research assistants were trained for seven days in March 2015. Nine research teams were formed, with three teams per senatorial zone. In each zone, the teams obtained data from hospitals and primary healthcare facilities applying the same selection criteria as in 2012 and yielding the same sample size. Research assistants obtained data through individual interviews of the heads of facilities (managers) and health service providers and from the facilities’ records—including registers of labor and childbirth, partographs, the operating room, and the prenatal and postnatal wards. Data on maternal complications and deaths at each facility were collected from Health Management Information System (HMIS) data registers on a monthly basis for 12 months (October 2013 to September 2014). Direct observations were also carried out to determine availability of the basic, essential infrastructure, drugs, and supplies required to perform the signal functions.

### Data management and analysis

Data collected was captured into Epi Info version 7 (Centers for Disease Control and Prevention, Atlanta, GA, USA) by trained data clerks. Descriptive analyses including frequency distributions and bivariate analyses were performed with SPSS version 15 (SPSS Inc, Chicago, IL, USA).

### Data quality assurance

The unique identification numbers assigned to facilities at baseline were used in this study to trace each facility. The list of health care facilities with their codes was shared with the data collectors and supervisors during the training. Data collectors were required to carefully check all recorded responses and correct any possible errors. Study supervisors reviewed administered questionnaires on a daily basis and probed for and addressed data inconsistencies. The survey coordinator at the state level performed a second level review of the administered questionnaires. Data entry queries were run to identify any issue regarding inconsistent or missing information. Specific questionnaires were reviewed for the necessary corrections. In addition to using queries to detect data entry errors, the design of the data entry screen also included in-built validation measures.

### Ethical approval

Ethical clearance for this study was granted by the Bauchi State Health Research and Ethics Committee (BHREC). Written informed consents were also obtained from the heads of the health facilities before the interviews and from patients for care that was observed. All data was anonymized and processed with the strictest confidentiality.

## Results

Given Bauchi State’s estimated population of 6,318,297 and based on the minimum acceptable level of EmOC [15], there ought to be at least 63 fully functioning EmOC facilities, 13 of which should be comprehensive.

### Availability of EmOC services

In 2012, just six (10.2%) of the 59 sampled facilities met the UN requirements for EmOC centers. By 2015, the number had increased to 21 (35.6%, p<0.001). The number of health facilities fully functioning as basic EmOC (BEmOC) increased from three of the total 59 (5.1%) in 2012 to eight (13.6%, p<0.002) in 2015. The number of facilities that fully functioned as comprehensive emergency obstetric centres (CEmOC—performed all prescribed nine signal functions—in the 3-month period preceding the survey) rose from 3 (5.1%) of the designated 21 hospitals in 2012 to 13 (22.0%, p<0.001) in 2015 (Table 1). Seven (18.4%) of the designated 38 health centers met the criteria for basic EmOC in 2015, while only one (2.6%) provided all the seven BEmOC signal functions in 2012 (p<0.05). None of the 3 senatorial zone met minimum standard for at least five emergency obstetric care facilities (including at least one comprehensive facility) for every 500, 000 population.

**Table 1:** Availability of EmONC by type of facility, Bauchi, Nigeria 2012 and 2015^Ω^.

	2012	2015	P-value	2012	2015	P-value	2012	2015	P-value
Type of EmOC provided	All facilities (n = 59) (%)	All facilities (n = 59) (%)		Hospitals (n = 21) (%)	Hospitals (n = 21) (%)		Health centers (n = 38) (%)	Health centers (n = 38) (%)	
At least 7 BEmOC signal functions provided	3 (5.1)	8 (13.6)	0.0018	2 (9.5)	1 (4.8)	0.0122	1 (2.6)	7 (18.4)	0.0561
All 9 CEmOC signal functions provided	3 (5.1)	13 (22.0)		3 (14.3)	13 (61.9)		0 (0.0)	0 (0.0)	
Facilities not providing all EmOC signal functions for their designated level of care	53(89.8)	38 (64.4)		16 (76.2)	7 (33.3)		37 (97.4)	31 (81.6)	

Abbreviation- EmOC: emergency obstetric care.

^Ω^Values are given as number and/or percentage. P-value generated using Fisher's exact test

**Fig 1:**
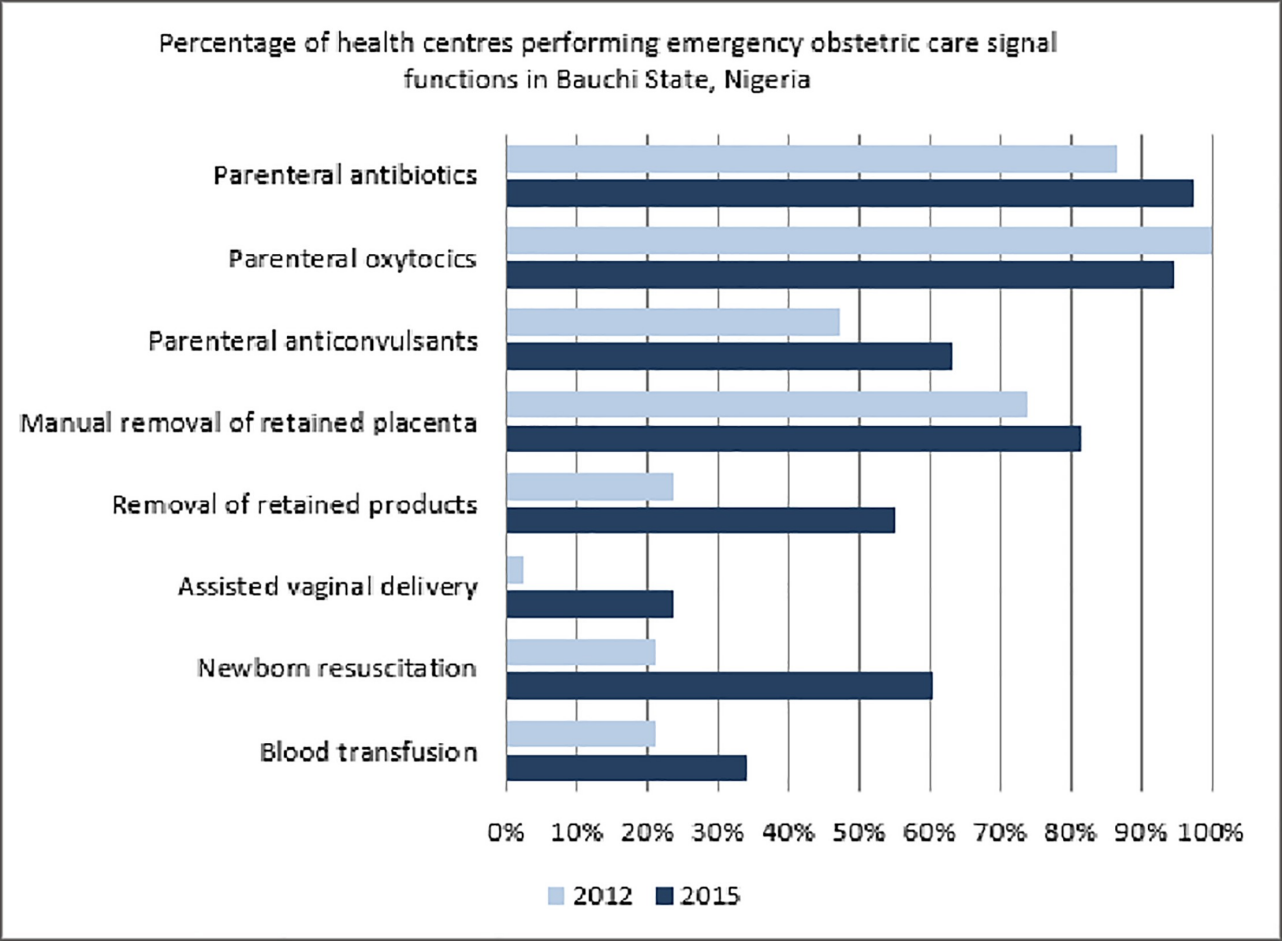
Signal functions provided by health centers.

All the hospitals administered parenteral antibiotics, gave uterotonic drugs and performed blood transfusion in 2012 and 2015 respectively. In addition, all except one hospital administered anticonvulsants, performed manual removal of placenta and the evacuation of retained products of conception in 2015 (Table 2). The number of hospitals that performed neonatal resuscitation in the 3-month period preceding the survey increased from 16 (76.2%) in 2012 to 21 (100%, p = 0.5113) in 2015. However, the number of hospitals that conducted cesarean births increased from 16 (76.2%) in 2012 to only 17 (81.0%) in 2015 (Fig 1). Assisted vaginal birth, a signal function that requires specific equipment and skills, was the least performed signal function in the hospitals though this demonstrated an increase in the number of hospitals performing the procedure from only six (28.6%) in 2012 to 14 (66.7%, p = 0.1153) in 2015 (Table 2).

**Table 2:** Availability of EmONC by type of facility, Bauchi, Nigeria 2012 and 2015 ^Ω^.

					Type of facility					
	Hospital					Primary Health care facilities				
	2012 (n = 21)		2015 (n = 21)		P-Value	2012 (n = 38)		2015 (n = 38)		P-Value
Signal Function	n	%	n	%		n	%	n	%	
Parenteral Antibiotics	21	100.0%	21	100.0%	1.1222	33	86.8%	37	97.4%	0.7202
Parenteral Oxytocics	21	100.0%	21	100.0%	1.1222	38	100.0%	36	94.7%	0.9075
Parenteral Anticonvulsants	21	100.0%	20	95.2%	1.0000	18	47.4%	24	63.2%	0.4407
Manual Removal of Placenta	21	100.0%	20	95.2%	1.0000	28	73.6%	31	81.6%	0.7948
Removal of Retained Products	19	90.5%	20	95.2%	1.0000	9	23.7%	21	56.3%	0.0427
Assisted Vaginal Delivery	6	28.6%	14	66.7%	0.1153	1	2.6%	9	23.7%	0.0214
Neonatal Resuscitation	16	76.2%	21	100.0%	0.5113	8	21.1%	23	60.5%	0.0106
Blood Transfusion	21	100.0%	21	100.0%	1.1222	8	21.1%	13	34.2%	0.3836
Surgery / Cesarean	16	76.2%	17	81.0%	1.0000	N/A		N/A		N/A

^Ω^Values are given as number and/or percentage. P-value generated using Fisher's exact test

Probability significance: P>.10 indicates non-significant, 0.05<p<10 indicates a tendency to be significant, P < .05 indicates significant at alpha = 0.05 and P < .01indicates significant at alpha = 0.01

**Fig 2:**
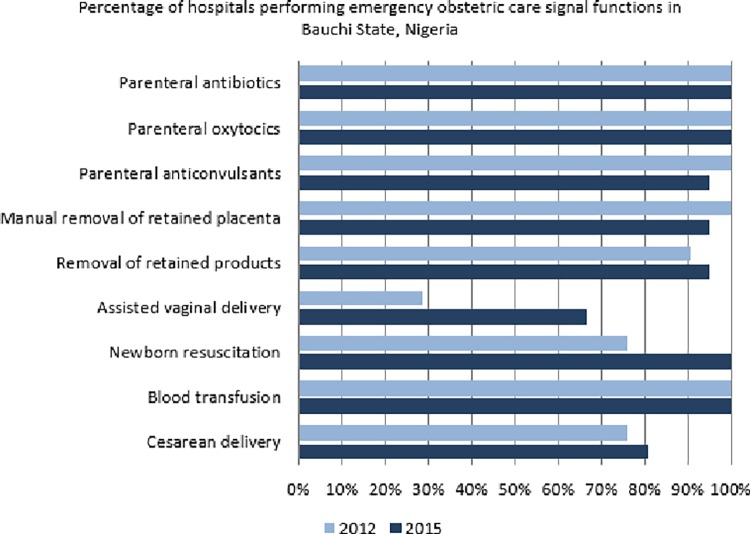
Signal functions provided by general hospitals.

An increase in provision of all signal functions for basic EmOC with the exception of administration of parenteral uterotonics was observed in all health centers. The number of health centers that administered anticonvulsants increased from 18 (47.4%) in 2012 to 24 (63.2%, p = 0.4407) in 2015 (Table 2). Significant improvement from baseline was recorded in performing those signal functions requiring both manual skills and specific equipment. Removal of retained products of conception (p <0.05) and assisted vaginal birth (p <0.05) were performed in 21 (56.3%) and 9 (23.7%) health centers in 2015 compared to only 9 (23.7%) and 1 (2.6%) in 2012 respectively (Fig 2). Health centers that performed neonatal resuscitation increased from eight (21.1%) in 2012 to 23 (60.5%) in 2015 (p<0.01). Blood transfusion service, a CEmOC signal function, was available in eight (21.1%) and 13 (34.2%) health centers in 2012 and 2015 respectively (Table 2).

Multiple responses were obtained from the healthcare workers on the reasons for non-performance of signal functions in hospitals and PHCs. Lack of equipment and supplies mentioned in 47.4% in 2012 declined to 26.6% in 2015. Lack of training as a reason for not performing signal functions equally dropped from 34.9% in 2012 to 29.6% in 2015. However, absence of indication for a particular signal function increased from 14.0% in 2012 to 28.8% in 2015.

### Utilization of EmOC services

The total population served was 5,615,369 in 2012 and 6,204,515 in 2015 with expected number of births in all health facilities of 232,913 in 2012 and 241,976 in 2016 respectively.

Overall, 19,383 or 8.0% (95% Confidence Interval, CI: 7.8% to 8.2%) of the expected population-based annual births took place in EmOC facilities in 2015 compared to 8,483 or 3.6% (95% CI: 3.5% to 3.8%) recorded in 2012. The proportion of births that occurred in all surveyed facilities combined also increased from 12.2% (95% CI: 11.9% to 12.5%) in 2012 to 14.8% (95% CI: 14.5% to 15.1%) in 2015. A decline of institutional births from 8.6% (95% CI: 8.4% to 8.9%) in 2012 to 6.8% (95% CI: 6.7% to 6.9%) in 2015 was observed in non-EmOC facilities (Table 3).

**Table 3:** Deliveries and complications in EmONC facilities, by zone, by year, Bauchi, Nigeria 2012 and 2015 (%) ^Ω^.

	State		South Zone		Central Zone		North Zone	
Year	2012	2015	2012	2015	2012	2015	2012	2015
Total population	5,715,292	6,318,297	2,096,472	2,317,666	1,770,140	1,956,902	1,848,680	2,043,729
Catchment Population	5,615,369	6,204,515	1,993,549	2,203,883	1,770,140	1,956,902	1,848,680	2,043,729
**Births:**								
Number expected in all Facilities	232,913	241,976	82,732	85,951	73,461	76,319	76,720	79,705
Number attended in EmOC facilities (%)	8,483 (3.6)	19,383 (8.0)	5,565 (6.7)	9,951 (11.6)	2,204 (3.0)	6,649 (8.7)	714 (0.9)	2,783 (3.5)
Confidence Interval	+/-0.15%	+/-0.22%	+/-0.34%	+/-0.43%	+/-0.25%	+/-0.40%	+/-0.14%	+/-0.25%
Number attended in non EmOC facilities (%)	19,972 (8.6)	16,435 (6.8)	5,863 (7.1)	4,928 (5.7)	8,177 (11.1)	6,933 (9.1)	5,932 (7.7)	4,574 (5.7)
Confidence Interval	+/- 0.25%	+/- 0.12%	+/- 0.35%	+/-0.31%	+/- 0.45%	+/- 0.41%	+/- 0.38%	+/- 0.32%
Total facility birth (%)	28,455 (12.2)	35,818 (14.8)	11,428 (13.8)	14,879 (17.3)	10,381 (14.1)	13,582 (17.8)	6,646 (8.7)	7,357 (9.2)
Confidence Interval	+-0.27%	+/-0.28%	+/- 0.47%	+/-0.51%	+/-0.50%	+/-0.54%	+/- 0.40%	+/- 0.40%
**Cesarean births:**								
Number in EmOC facilities (% of deliveries)	319 (3.8)	1,077 (5.6)	0 (0.0)	521 (5.2)	240 (10.9)	417 (6.3)	79 (11.1)	139 (5.0)
Confidence Interval	+/- 0.81%	+/-0.65%	+/- 0.07%	+/- 0.88%	+/- 2.60%	+/- 1.17%	+/-4.61%	+/- 16.25%
Number in EmOC facilities (% of expected deliveries)	319 (0.1)	1,077 (0.4)	0 (0.0)	521 (0.6)	240 (0.3)	417 (0.5)	79 (0.1)	139 (0.2)
Confidence Interval	+/- 0.03	+/- 0.05	+/- 0.00	+/- 0.10	+/- 0.08	+/- 0.10	+/- 0.05	+/- 0.06
**Complications:**								
Total expected number of direct complications	34,937	36,296	12,410	12,893	11,019	11,448	11,508	11,956
Direct complications treated in EmOC facilities (met need) (%)	1,167 (3.3)	3,608 (9.9)	284 (2.3)	1,116 (8.7)	641 (5.8)	2,030 (17.7)	247 (2.1)	466 (3.9)
Confidence Interval	+/- 0.38%	+/-0.62%	+/-0.53%	+/-0.97%	+/-0.87%	+/-1.40%	+/-0.53%	+/-0.69%
Direct complications treated in non-EmOC facilities (%)	2,888 (8.3)	1,145 (3.2)	1,048 (8.4)	351 (2.7)	1,894 (17.2)	345 (3.0)	969 (8.4)	449 (3.8)
Confidence Interval	+/-0.58%	+/-0.36%	+/-0.98%	+/-0.56%	+/-1.41%	+/-0.63%	+/-1.01%	+/-0.68%
Number of direct complications treated in facilities (%)	4,055 (11.6)	4,753 (13.1)	1,332 (10.7)	1,467 (11.4)	2,535 (23.0)	2,375 (20.7)	1,216 (10.6)	915 (7.7)
Confidence Interval	+/-0.67%	+/-0.69%	+/-1.09%	+/-1.10%	+/-1.57%	+/-1.49%	+/-1.12%	+/-0.95%

Abbreviation- EmOC: emergency obstetric care.

^Ω^Values are given as number and/or percentage.

The caesarean birth rate as a proportion of facility-based births occurring in EmOC facilities increased from 3.8% (95% CI: 3.0% to 4.6%) in 2012 to 5.6% (95% CI: 5.0% to 6.3%) in 2015. However, the overall population-based caesarean birth rate remained as low as 0.1% in 2012 and 0.4% in 2015. The rates did not differ significantly between the senatorial zones (Table 3).

Met need for EmOC is the proportion of women with direct obstetric complications treated in EmOC facility as a proportion of expected number of women who would have major obstetric complications, or 15% of expected births, during the same period in a specified area. The met need for EmOC increased significantly from 3.3% (95% CI: 2.9% to 3.7%) in 2012 to 9.9% (95% CI: 9.3% to 10.5%) in 2015. The proportion of women managed for obstetric complications in non-EmOC facilities dropped from 8.3% (95% CI: 7.7% to 8.9%) in 2012 to as low as 3.2% (95% CI: 2.8% to 3.6%) in 2015. This could be associated with increased number of EmOC facilities and improved referrals. Though met need for EmOC varied by zone, the same trends were observed in the three senatorial zones (Table 3).

Overall, 11,421 of the expected 34,937 complications were documented in all the study facilities in 2012, 4,055 (35.5%) of which were direct obstetric complications. The total number of complications declined to 9,060 of the expected 36,296 in 2015. However, the proportion of direct obstetric complications increased to 4,753 (52.2%).

Three (75.6%) in 4 women with direct obstetric complications in 2015 were treated in EmOC facilities compared to only 1 of 4 (24.4%) in 2012 (Table 3). In EmOC facilities, obstetric haemorrhage accounted for 349 or 29.9% (95% CI: 27.3% to 32.5%) and 1,365 or 37.8% (95% CI: 36.2% to 39.4%) of obstetric direct complications recorded in 2012 and 2015 respectively. Obstructed/prolonged labor as a proportion of direct complications declined from 10.4% (95% CI: 8,7% to 12.2%) to 8.8% (95% CI: 7.9% to 9.7%) while complications of abortion dropped significantly from 17.1% (95% CI: 14.9% to 19.3%) to 6.9% (95% CI: 6.1% to 7.7%) between 2012 and 2015 respectively. No difference was observed in the proportion of women with pre-eclampsia/eclampsia in 2012 28.2% (95% CI: 25.6% to 30.8%) and 2015 29.6% (95% CI: 28.1% to 31.1%).

Among the 7,366 women with indirect complications in 2012 and 4,307 in 2015, malaria was responsible for 68.4% and 39.0% respectively. Severe anemia accounted for 30.8% in 2012 and 59.9% in 2015 of all the indirect complications.

### Quality of EmOC services

The direct obstetric case fatality rate (CFR) is a proxy indicator for the changes of quality of care. An increase of CFR in the EmOC facilities from 3.1% (95% CI: 2.1% to 4.1%) in 2012 to 4.0% (95% CI: 3.5% to 4.7%) in 2015 was observed (Table 4). Higher patient load, late arrival and better record keeping may explain the associated increase in case fatality rate.

**Table 4:** Obstetric complications treated and direct and indirect causes of maternal deaths in sampled health facilities in Bauchi State, Nigeria^Ω^.

	EmOC facilities				Non-EmOC Facilities			
	2012		2015		2012		2015	
Type of complications	Number of women affected (%)	Number of deaths (%)	Number of women affected (%)	Number of deaths (%)	Number of women affected (%)	Number of deaths (%)	Number of women affected (%)	Number of deaths (%)
***A*. *Direct causes***	1167 (100.0%)	36 (3.1%)	3608 (100.0%)	143 (4.0%)	2888 (100.0%)	246 (8.5%)	1145 (100.0%)	57 (5.0%)
***B*.**		+/-1.00%		+/-0.64%		+/-1.02%		+/-1.27%
Hemorrhage (APH/PPH)	349 (29.9%)	3 (8.3%)	1365 (37.8%)	39 (27.3%)	112 (3.9%)	49 (19.9%)	252 (22.0%)	13 (22.8%)
CI	+/- 2.62%	+/-9.48%	+/-1.58%	+/-7.23%	+/- 0.71%	+/- 4.97%	+/- 2.40%	+/- 10.68%
Retained placenta	85 (7.3%)	0 (0.0%)	275 (7.6%)	7 (4.9%)	554 (19.2%)	4 (1.6%)	137 (12.0%)	3 (5.3%)
CI	+/- 1.50%	+/-4.82%	+/-0.87%	+/-3.68%	+/- 1.44%	+/- 1.74%	+/- 1.88%	+/-6.28%
Obstructed/ prolonged labor	121 (10.4%)	7 (19.4%)	319 (8.8%)	21 (14.7%)	284 (9.8%)	16 (6.5%)	61 (5.3%)	16 (28.1%)
CI	+/- 1.75%	+/-12.64%	+/-0.93%	+/-5.80%	+/- 1.09%	+/- 3.13%	+/- 1.31%	+/-11.38%
Ruptured uterus	14 (1.2%)	1 (7.1%)	16 (0.4%)	0 (0.0%)	165 (5.7%)	1 (0.4%)	1 (0.1%)	0 (0.0%)
CI	+/- 0.74%	+/-6.84%	+/-0.22%	+/-1.31%	+/- 0.85%	+/- 1.10%	+/- 0.24%	+/-3.16%
Severe pre-eclampsia / eclampsia	329 (28.2%)	21 (58.3%)	1068 (29.6%)	40 (28.0%)	1151 (39.9%)	128 (52.0%)	165 (14.4%)	6 (10.5%)
CI	+/- 2.58%	+/-15.33%	+/-1.49%	+/-7.28%	+/- 1.78%	+/- 6.19%	+/- 2.03%	+/-8.10%
Complications of abortion	199 (17.1%)	1 (2.8%)	249 (6.9%)	2 (1.4%)	365 (12.6%)	1 (0.4%)	239 (20.9%)	17 (29.8%)
CI	+/- 2.16%	+/-6.84%	+/-0.83%	+/-2.29%	+/- 1.21%	+/- 1.10%	+/- 2.35%	+/- 11.57%
Ectopic pregnancy	2 (0.2%)	0 (0.0%)	4 (0.1%)	1 (0.7%)	6 (0.2%)	0 (0.0%)	0 (0.0%)	0 (0.0%)
CI	+/- 0.29%	+/-4.82%	+/- 0.12%	+/-1.87%	+/- 0.18%	+/- 0.77%	+/- 0.29%	+/- 3.16%
Other	68 (5.8%)	3 (8.3%)	312 (8.6%)	33 (23.1%)	251 (8.7%)	47 (18.7%)	290 (25.3%)	2 (3.5%)
	+/- 1.35%	+/- 9.48%	+/- 0.92%	+/-6.85%	+/- 1.03%	+/- 4.90%	+/- 2.52%	+/- 5.48%
***C*. *Indirect causes***	740 (100.0%)	6 (0.8%)	2654 (100.0%)	53 (2.8%)	6626 (100.0%)	55 (0.8%)	1653 (100.0%)	29 (1.8%)
Malaria	202 (27.3%)	0 (0.0%)	633 (23.9%)	2 (6.8%)	4835 (73.0%)	4 (7.3%)	1046 (63.3%)	12 (41.4%)
CI	+/- 3.20%	+/- 19.52%	+/- 1.62%	+/- 5.86%	+/- 1.07%	+/- 7.20%	+/- 2.32%	+/- 16.87%
Severe Anemia	526 (71.1%)	6 (100.0%)	1979 (74.6%)	49 (92.5%)	1740 (26.3%)	49 (89.1%)	599 (36.2%)	17 (58.6%)
CI	+/- 3.26%	+/- 19.52%	+/- 1.66%	+/- 7.44%	+/- 1.06%	+/- 8.36%	+/- 2.31%	+/- 16.87%
Diabetes mellitus	0 (0.0%)	0 (0.0%)	11 (0.4%)	0 (0.0%)	0 (0.0%)	0 (0.0%)	2 (0.1%)	0 (0.0%)
CI	+/- 0.26%	+/- 19.52%	+/- 0.25%	+/- 3.38%	+/- 0.03%	+/- 3.26%	+/- 0.20%	+/- 5.85%
Hepatitis	0 (0.0%)	0 (0.0%)	1 (0.0%)	0 (0.0%)	0 (0.0%)	0 (0.0%)	0 (0.0%)	0 (0.0%)
CI	+/- 0.26%	+/- 19.52%	+/- 0.10%	+/- 3.38%	+/- 0.03%	+/- 3.26%	+/- 0.12%	+/- 5.85%
Other indirect complications	12 (1.6%)	0 (0.0%)	30 (1.1%)	2 (3.8%)	51 (0.8%)	2 (3.6%)	6 (0.4%)	0 (0.0%)
CI	+/- 0.94%	+/- 19.52%	+/- 0.41%	+/- 5.86%	+/- 0.21%	+/- 5.66%	+/- 0.31%	+/- 5.85%

Abbreviation: EmOC, emergency obstetric care.

^Ω^Values are given as number and/or percentage.

Major direct obstetric complications were responsible for 282 (82.2%) of the total 343 and 200 (70.9%) of the total 282 maternal deaths from direct and indirect obstetric complications recorded in 2012 and 2015 respectively. Indirect obstetric complications contributed to 17.8% and 29.1% of maternal deaths in 2012 and 2015 respectively. A reduction in the proportion of maternal deaths due to pre-eclampsia/eclampsia from 58.3% (95% CI: 43.0% to 73.6%) to 28.0% (95% CI: 20.7% to 30.3%) was recorded between 2012 and 2015 in all the study facilities (Table 4). This can be attributed to training and availability of magnesium sulphate for prevention and management of eclampsia. Obstetric haemorrhage was responsible for one-fifth of maternal deaths due to direct complications in 2012 and one-quarter in 2015 respectively. Severe anaemia was responsible for 55 (90.2%) of 61 deaths attributable to indirect causes in 2012 and 66 (80.5%) of 82 deaths in 2015 respectively.

## Discussion

Findings from this study indicated improvement in availability, utilization and quality of EmOC services in Bauchi state between 2012 and 2015. However, this is far below minimum acceptable standard by UN process indicators.

Given Bauchi State’s estimated population of 6,318,297 and based on the minimum acceptable level of EmOC [15], it managed to have just 21 out of the expected minimum 63 fully functioning EmOC facilities in 2015. This increase in the number of facilities that met the criteria for BEmOC from 3 to 8 in 2015 represented only 16.0% of the minimum acceptable level for basic EmOC facilities for Bauchi State. The state succeeded in attaining the minimum expected 13 functional CEmOC, which increased from 3 in 2012. In effect, the State achieved the minimum acceptable level for CEmOC of at least 1/500,000 population. There remains a critical need for deliberate and concerted efforts to increase the number of health centers with strengthened capacity to provide BEmOC, which is essential to improving access and use of quality EmOC [8,10,16].

An insufficient number of facilities that meet the criteria for BEmOC with corresponding adequate number of fully functioning CEmOC facilities as reported in our study is comparable to findings of EmOC surveys in similar low resource settings [10,17–21]. Kongnyuy *et al* however, reported no change in the number of comprehensive and basic EmOC facilities over a three year period in Malawi [16], underscoring several challenges stakeholders face in the improvement of availability, access and use of quality EmOC, particularly in low resource settings [22]. While our study and most of the EmOC studies conducted in developing country settings with high maternal mortality and morbidity involved public health facilities only, Saidu et al [22] reported that more facilities in the private sector meet the criteria for EmOC compared with public sector in similar settings. This indicates the important role and contribution private health facilities can play and provide for improved access and quality of EmOC.

Our study found significant increase for provision of those signal functions that required specific equipment and skills, including assisted vaginal delivery, removal of retained products of conception and neonatal resuscitation in both hospitals and health centers. Studies in similar settings have however indicated poor performance of the signal functions, particularly in health centers [10,18]. This shows that a suite of interventions, including facility improvement, competency-based training, and provision of specific equipment is essential in strengthening the capacity of health service providers and facilities to provide quality EmOC. The number of hospitals conducting cesarean delivery changed little despite targeted interventions. This could be associated with the non-availability of additional doctors.

Lack of supplies and training, and absence of indication to conduct of a particular intervention as major reasons for non-performance of signal functions reported in our study are comparable with findings reported by Echoka *et al* in similar settings in Kenya [23].

The increased proportion of births from 3.6% in 2012 to 8.0% in 2015 recorded in EmOC facilities is lower than the recommended minimum of 15% by UN calling for demand generation activities.

In spite of adequate number of CEmOC facilities, the population-based caesarean birth rates of 0.1% in 2012 and 0.4% in 2015 also were well below the minimum acceptable level of 5.0% [15] and rates recorded in similar settings [10,23–27]. This can partly be attributed to inadequate number of medical doctors in CEmOC facilities, low demand generation, poor emergency transportation resulting to low access to CEmOC facilities.

Even though the met need for EmOC services more than doubled (from 3.4% in 2012 to 10.0% in 2015), it is well below UN target of 100% and findings reported in EmOC studies of similar settings [14,24,25,28]. Met need for EmOC of 10.0% reported in our study together with 8.0% of births in EmOC facilities indicates low utilization of health facilities for EmOC in the state, and is in keeping with the association between met need and the proportion of births attended by skilled birth professionals reported by Holmer *et al* [14].

The number of direct obstetric complications treated in EmOC facilities (met need) in 2015 increased by more than 300% compared with 2012. However, this increase in utilization was also associated with an increase in direct obstetric case fatality rate (DOCFR) from 3.1% in 2012 to 4.0% in 2015. While this increase in DOCFR is lower than reported by Otchere *et al* in Mali [26], it remains higher than <1% acceptable by UN and the findings reported in EmOC studies of similar settings [24,25,28]. The rise in DOCFR reported in our study could be associated with possible late arrivals to facilities, increased number of women treated in EmOC facilities, improved record keeping and reporting.

Major direct obstetric complications were responsible for 70.9% of the total maternal deaths from direct and indirect obstetric complications recorded 2015, lower than 80.1% recorded in 2012 and 96.0% reported by Kim *et al* [27].

Whereas the reported number of women treated for haemorrhage increased from 11.4% in 2012 to 34.0% in 2015, there was little change in the proportion of maternal deaths due to hemorrhage. This could be associated with increased awareness leading to higher recognition and diagnosis, and improved referrals. While proportion of women with pre-eclampsia/eclampsia changed little from 28.2% in 2012 to 29.6% in 2015, significant decrease in attributed maternal deaths from 52.8% to 23.0% was observed in 2012 and 2015 respectively. Anaemia and malaria remain the leading indirect complications and causes of maternal deaths similar to findings reported in other studies from low resource countries [8,20]. This necessitates review of the current preventive measures for anaemia and malaria at facility and community levels.

### Lessons learnt

Many challenges were encountered in the course of supporting Bauchi state to improve the health systems for increased access and utilization of quality EmOC services. These included inadequate funding, shortage of Human Resources for Health and inequitable distribution of those available. This was a major challenge, particularly for PHCs to achieve full BEmOC status. Other challenges were poor infrastructural maintenance culture, minimal constructive engagement with communities in planning, implementation and monitoring health interventions in order to foster real community participation among others. These challenges are critical for sustainability of current efforts and future planning by the state and implementing partners.

Identifying and strengthening existing government structures is key to ensuring sustainability, supportive supervision provided unique opportunity to assess knowledge and skills retention and re-enforce good practice, identify gaps and develop action plans to address gaps. Government support and collaboration provided an enabling environment for improving the quality of health services and promoted synergy and a platform for ensuring sustainability.

On a positive note is the enactment of the country’s National Health Act (NHA) in 2014, which commenced implementation in 2018 with a budget of approximately $181 million. This act provides for a Basic Health Care Provision Fund (BHCPF) largely from the Government of Nigeria resources and is the country’s clear statement of intent to take responsibility for the health of its citizens. Fifty percent of the funds have been earmarked to be used for the provision of a basic minimum package of health services to citizens, in eligible 'primary or secondary health care facilities through the National Health Insurance Scheme (NHIS). Furthermore, 20 per cent of the Fund is earmarked to be used to provide essential drugs, vaccines and consumables for eligible primary health care facilities while 15 per cent of the Fund will be used for the provision and maintenance of facilities, equipment and transport for eligible primary healthcare facilities. Finally, 10 per cent of the Fund will be used for the development of human resources for primary health care while the remaining 5 per cent of the fund will be used for emergency medical treatment. Bauchi State is eligible to draw down from the BHCPF in order to replicate all that was achieved under the TSHIP program. Many development partners have also committed to donate to the BHCPF basket to sustain efforts to improve maternal and newborn health care in the country.

This study has few limitations. Use of convenience sampling to select primary healthcare centers clustered around the hospitals in the two surveys does limit generalization of the findings. However, pre-defined selection criteria were used to minimize any bias. Private health facilities were not included in the studies despite their potential roles in the provision of EmOC services, particularly in underserved communities. Those attending such facilities are generally of a higher wealth quintile and may differ in characteristics and health seeking behavior from those attending public health facilities. The findings of the study have however, provided important lessons learned, issues and challenges that will go a long way in scaling up efforts to improve the quality of EmOC services in Bauchi State. The findings can also be used to justify the operationalization of the national Task Shifting and Task Sharing Policy that allows the training of Community Health Extension Workers (CHEWs) and Community Health Officers (CHOs) to perform additional signal functions.

## Conclusion

Availability and utilization of quality emergency obstetric services still remain a challenge in Bauchi state, Nigeria. However, the demonstrated improvements recorded in proportion of births and met need for EmOC suggest that despite the challenges, the interventions supported and strengthened the health system’s capacity to improve the availability, access and utilization of EmOC services in Bauchi State. The rise in case fatality rate reported in our study merits further study, and new strategies to reduce late arrival of women with life-threatening complications to health facilities. The study also showed an increased number of women treated in EmOC facilities and improved record keeping and reporting. There is an urgent need for collaborative efforts between Federal Government of Nigeria, Bauchi State Government, health related agencies, donor organizations, training institutions and communities to continue strengthening health facilities to meet EmOC standards, with particular attention to BEmOC services in the state.
